# Rezafungin for sternal wound infection caused by multidrug-resistant Candidozyma auris: a case report

**DOI:** 10.1099/acmi.0.001171.v4

**Published:** 2026-11-12

**Authors:** Suvitesh Luthra, Annette David, David Thirukumaran, Tat Shing Yam

**Affiliations:** 1 University Hospital Southampton National Health Service Foundation Trust, Southampton, UK

**Keywords:** *Candidozyma auris*, echinocandin, multidrug resistance, outpatient antifungal therapy, rezafungin, sternal wound infection

## Abstract

**Introduction.** *Candidozyma auris* is an emerging multidrug-resistant fungal pathogen associated with healthcare outbreaks and significant mortality. Management can be challenging due to antifungal resistance and the need for prolonged intravenous therapy.

**Case Presentation.** A 58-year-old man developed a postoperative sternal wound infection caused by multidrug-resistant *C. auris* following aortic valve replacement, coronary artery bypass grafting and ascending aortic interposition graft surgery. His postoperative course was complicated by *Staphylococcus epidermidis* prosthetic graft infection requiring redo valve replacement and graft repair and placement of vacuum-assisted closure. Two weeks after discharge home, persistent wound discharge yielded *C. auris*. Following surgical debridement and initiation of intravenous caspofungin with oral itraconazole, once-daily intravenous antifungal therapy was transitioned to once-weekly intravenous rezafungin to facilitate outpatient treatment coordinated with vacuum-assisted wound therapy.

**Conclusion.** After 12 weeks of dual antifungal therapy, complete clinical and microbiological resolution was achieved without adverse events. This case highlights the feasibility of rezafungin as an outpatient treatment strategy for complex multidrug-resistant *C. auris* infections.

## Data Summary

All relevant clinical and microbiological data supporting this case report are included within the manuscript. No genomic datasets were generated. De-identified clinical data are available from the corresponding author upon reasonable request.

## Introduction

*Candidozyma auris* is an emerging yeast pathogen responsible for healthcare-associated outbreaks and invasive infections worldwide [1]. Since its first description in 2009 and subsequent taxonomic reclassification in 2023, the organism has demonstrated multidrug resistance, environmental persistence and the ability to colonize hospital environments [1, 2]. Mortality associated with invasive infection has been reported to range between 30% and 60%, particularly among critically ill individuals [3]. Management of infection is complicated by resistance to multiple antifungal classes and the need for prolonged intravenous therapy [1]. Echinocandins are recommended as first-line therapy for *C. auris* infection [1, 2]. However, daily intravenous administration may limit outpatient management [4]. Rezafungin is a next-generation echinocandin with a prolonged elimination half-life of ~133 h, enabling once-weekly dosing while maintaining therapeutic drug concentrations [4, 5].

## Case presentation

### Patient information

A 58-year-old man with hypertension, hypercholesterolaemia, psoriasis, psoriatic arthritis and type 2 diabetes mellitus underwent aortic valve replacement using a 27-mm Inspiris Resilia prosthetic valve, coronary artery bypass grafting using a saphenous vein graft to the left anterior descending artery and interposition graft replacement of the ascending aorta using a 32-mm vascular graft (Table 1). He was routinely admitted 1 day prior to his elective surgery .

**Table 1.** Clinical timeline

Date	Clinical course	Microbiological finding	Management
**08 May 2024**	Aortic valve (27 mm Inspiris Resilia) replacement, coronary artery bypass grafting and ascending aortic interposition graft	–	Surgical intervention, 3 doses of cefuroxime were given as routine antibiotics policy of the institution for patient undergoing valve surgery.
**11 May 2024**	Postoperative fever	–	Empiric intravenous vancomycin was initiated.
**23 May 2024**	Persistent pyrexia and anaemia	*S. epidermidis* isolated from peripheral blood, central venous catheter and mediastinal drain fluid	Blood transfusion; vancomycin was stopped, escalation to piperacillin–tazobactam and oral doxycycline for 5 days, Daptomycin was started; Peripherally inserted central catheter line was replaced.
**27 May 2024**	Cardiac tamponade requiring redo sternotomy	*S. epidermidi*s DNA detected in peri-graft haematoma by PCR (culture negative)	Surgical drainage, patient was continued on daptomycin and treated as early postoperative infective endocarditis.
**25 June 2024**	Redo tissue aortic valve replacement (27 mm Magna Ease) and graft repair, VAC dressing	Explanted prosthetic valve culture positive for *S. epidermidis*	Surgical intervention
**Late June–July 2024**	Postoperative recovery	–	Patient continued to have daptomycin and started oral rifampicin (6 weeks).
**02 August 2024**	Recurrent pyrexia	Blood cultures (via peripherally inserted catheter line) positive for *C. koseri*	Patient was started on ertapenem and later discharged home on 05 August 2024 with oral ciprofloxacin till 07 August 2026 and doxycycline for 4 weeks.
**21 August 2024**	Persistent sternal wound discharge and readmission	Wound swab positive for *C. albicans* and *C. auris*; inflammatory markers – crp-14, normal white cells and neutrophils, mild lymphopenia	Surgical debridement with Vacuum associated closure dressing and antifungal therapy. 23 August 2026: Intravenous caspofungin 70 mg loading dose was administered during re-exploration and Vacuum associated closure insertion for sternal wound infection. 24 August 2026: caspofungin 50 mg once daily commenced and continued until 30 August 2026. 30 August 2026: caspofungin increased to 70 mg once daily; oral itraconazole was added, and serum levels were checked after 1 week. 03 September 2026: Computed Tomography of the chest was performed; osteomyelitis was excluded. 06 September 2026: Vacuum associated closure dressing changed in theatre; pre-dose itraconazole level checked; a loading dose of rezafungin 400 mg was administered and caspofungin was discontinued.
**07 September 2024**	Clinical improvement; discharge	–	07 September 2026: the patient was discharged home with a plan to continue rezafungin 200 mg intravenously once weekly as the maintenance dose (to coincide with weekly VAC dressing changes) and oral itraconazole 300 mg twice daily; the planned total duration of antifungal therapy is 3 months; weekly rezafungin with outpatient wound review
**12-week follow-up**	Complete wound healing	Negative cultures	Completion of antifungal therapy

### Postoperative course and bacterial infection

On postoperative day 3, the patient developed fever and sternal wound discharge, and intravenous vancomycin therapy was commenced.

Persistent pyrexia and haemoglobin decline prompted further investigation. Antibiotics were escalated to Piperacillin–tazobactam and doxycycline for 5 days, vancomycin was stopped and the patient was commenced on a course of daptomycin (Table 1).

The clinical course was complicated by cardiac tamponade requiring redo sternotomy and surgical decompression. Progressive graft infection and pseudoaneurysm subsequently required redo tissue aortic valve replacement and repair of the ascending aortic interposition graft.

Microbiological culture of the infected prosthetic aortic valve grew *Staphylococcus epidermidis*. The patient continued intravenous daptomycin, and oral rifampicin was started for a total duration of 6 weeks.

Later during admission, the patient developed pyrexia, blood cultures via peripherally inserted central catheter yielded *Citrobacter koseri* and the patient received intravenous ertapenem initially which was switched to oral ciprofloxacin.He was discharged on 05 August 2024 with a course of oral ciprofloxacin till 07 August 2024 and doxycycline for 4 weeks (Table 1).

Following this prolonged hospitalization, the patient was discharged home with weekly follow-up in the wound clinic for vacuum-assisted closure therapy.

### Development of fungal infection

Two weeks after discharge, the patient presented with a persistent brownish discharge from the sternal wound.

Computed tomography of the chest excluded mediastinitis and osteomyelitis. The wound appeared superficial, with slough and surrounding inflammation. The sternal wires remained *in situ* as there was no deep-seated infection.

Wound swab cultures grew *Candida albicans* and *C. auris*, and the patient was readmitted for operative wound assessment and management. *C. auris* was isolated from bone sample, and the organism was seen for more than a month; hence, the chance that it could be a potential contaminant was ruled out.

Co-infection was ruled out as all the sternal wound swabs including bone sample had *C. auris* and *C. albicans*.

### Operative management

The patient underwent surgical debridement with removal of necrotic tissue and replacement of the vacuum-assisted wound closure therapy dressing.

Tissue samples including muscle, bone and wound fluid were collected for microbiological analysis. Bone samples were positive for both *C. albicans* and *C. auris*.

### Diagnostic assessment

Microbiological cultures were performed using Columbia blood agar, cystine lactose electrolyte-deficient agar, Columbia nalidixic acid-selective blood, fastidious anaerobe agar with neomycin and Sabouraud dextrose agar.

Targeted screening for *C. auris* utilized CHROMagar Candida Plus media (EO Labs).

Species identification was performed using MALDI-TOF MS (MALDI Biotyper® Sirius, Bruker). No other method was used.

Isolates were sent to the UK Health Security Agency Mycology Reference Laboratory, Bristol, for confirmation.

MICs were determined using in-house E-test (epsilometer) strips (bioMérieux) according to Clinical and Laboratory Standards Institute (CLSI) guidelines, which were also used for interpretation of the MIC results. Interpretation for yeasts was based on CLSI species-specific breakpoints where available or on normal ranges where clinical breakpoints were not available (Table 2).

**Table 2.** Antifungal susceptibility profile

Antifungal agent	Interpretation	MIC
Amphotericin B	Susceptible	Not reported
Anidulafungin	Susceptible	Not reported
Flucytosine	Intermediate	Not reported
Fluconazole	Resistant	Not reported
Isavuconazole	Intermediate	Not reported
Itraconazole	Intermediate	Not reported
Posaconazole	Resistant	Not reported
Voriconazole	Intermediate	Not reported
Miconazole	Susceptible	0.125 mg l^−1^

### Antifungal management

Alongside surgical debridement, antifungal therapy was initiated with intravenous caspofungin for 2 weeks together with oral itraconazole (Table 2).

After clinical stabilization, therapy was transitioned to once-weekly intravenous rezafungin with an initial loading dose followed by weekly maintenance dosing. Oral itraconazole was continued throughout the 12-week duration. The decision to add oral itraconazole was based on the presence of exposed wire and the consequent risk of osteomyelitis. Itraconazole was considered appropriate given its favourable bone penetration. Furthermore, dual therapy was recommended by the Mycology Reference Unit.

Treatment administration was coordinated with scheduled wound clinic visits and vacuum-assisted wound therapy changes.

Laboratory monitoring including renal and hepatic function remained stable throughout therapy as well as therapeutic drug monitoring of itraconazole.

### Outcome and follow-up

After 12 weeks of antifungal therapy, the sternal wound had fully epithelialized. Inflammatory markers normalized, and repeated cultures were negative for *C. auris* and *C. albicans*.

No drug-related adverse events were observed, and the patient remained clinically well at 3-month follow-up.

### *C. auris* exposure and prevention measures

Owing to bed shortage, there were vascular outlier patients placed in the same open bay in the cardiothoracic ward as this patient. The vascular patients were subsequently screened positive for *C. auris* as part of the initial *C. auris* outbreak on the vascular ward around the end of July.

At that time, the patient had already been discharged home and found positive for *C. auris* in the sternal wound at a wound clinic visit. On readmission, he was isolated in a side room.

The patient was managed under isolation precautions with strict infection prevention and control measures. Daily environmental decontamination was performed using Actichlor disinfectant solution on all reusable equipment, and single-use devices such as blood pressure cuffs, stethoscopes and thermometers were utilized where possible. The patient also received decolonization and skin care with Octenisan wash and Octeniderm gel.

Routine environmental monitoring, including environmental swabbing of surfaces such as bed rails, was not performed as part of local practice. Environmental screening is undertaken only if specifically advised by the Infection Prevention and Control team, particularly in the setting of suspected clusters or outbreaks. Routine screening of contacts was not performed.

Terminal cleaning following patient discharge included chlorine-based disinfection, curtain replacement and UV light decontamination. In the event of patient clusters or outbreaks, hydrogen peroxide vapour cleaning protocols are implemented.

All vacuum-assisted wound therapy dressing changes were subsequently only performed in operating theatres or designated clinic rooms rather than in open ward bays to minimize the risk of cross-transmission.

## Discussion

This case illustrates a complex postoperative course involving sequential bacterial prosthetic graft infection followed by multidrug-resistant *C. auris* sternal wound infection.

Multiple factors likely contributed to fungal emergence including prolonged hospitalization, extensive antimicrobial exposure, repeat cardiac surgery and underlying metabolic and inflammatory comorbidities and exposure to a *C. auris* carrier [6].

Although echinocandins remain first-line therapy for *C. auris* infection, the requirement for daily intravenous administration may prolong hospitalization [7].

Rezafungin was chosen as the drug of choice to avoid further admission as the patient lived 60 miles away, outside our outpatient parenteral antimicrobial therapy catchment area. In addition, patient needed a weekly review and dressing of the sternal wound; hence, this provided the perfect opportunity for administration of the antifungal drug. Rezafungin is a novel echinocandin with an extended half-life that enables once-weekly dosing. In this case, weekly therapy allowed continuation of effective antifungal treatment while facilitating outpatient management [8, 9].[Bibr R3]

Further studies are required to clarify the optimal role and duration of rezafungin therapy in complex surgical fungal infections.

### Rezafungin vs caspofungin

Rezafungin’s major advantage is its very long half-life, allowing once-weekly dosing while maintaining efficacy comparable to daily caspofungin. While caspofungin remains the more established agent with broader clinical experience and additional approved indications (e.g. oesophageal candidiasis and salvage therapy for invasive aspergillosis). Current evidence shows similar efficacy and safety for candidemia/invasive candidiasis, with no significant differences in global cure or mortality [7, 10, 11] (Table 3)

**Table 3.** Differences between Caspofungin vs Rezafungin Table 3 [7, 10, 11, 13]

Feature	Caspofungin	Rezafungin
**Drug class**	Echinocandin	Next-generation echinocandin
**Mechanism of action**	Inhibits β-(1,3)-d-glucan synthase → inhibits fungal cell wall synthesis	Same mechanism; structural modification improves stability and pharmacokinetics
**Spectrum**	Candida *spp*. (fungicidal); Aspergillus *spp*. (fungistatic)	Similar spectrum; active against most Candida *spp*., including many azole-resistant isolates
**Administration**	Intravenous only	Intravenous only
**Standard dosing**	70 mg loading dose, then 50 mg daily (70 mg daily in selected patients)	400 mg on day 1, then 200 mg once weekly
**Half-life**	Approximately 9–11 h	Approximately 130–150 h (allows weekly dosing)
**Metabolism**	Slow peptide hydrolysis and N-acetylation; not Cytochrome P450-dependent	Minimal metabolism; highly stable; not Cytochrome P450-mediated
**Dose adjustment**	Reduce maintenance dose in moderate hepatic impairment; none for renal dysfunction	No dose adjustment for renal impairment; hepatic adjustment generally not required based on current labelling
**Approved indications**	Candidemia, invasive candidiasis, oesophageal candidiasis, salvage therapy for invasive aspergillosis	Adults with candidemia and invasive candidiasis (approval varies by region)
**Clinical advantages**	Extensive clinical experience; guideline-recommended first-line echinocandin	Once-weekly dosing, prolonged tissue exposure, potential outpatient therapy, less frequent intravenous access
**Limitations**	Daily intravenous administration; hepatic dose adjustment	Limited long-term clinical experience; higher acquisition cost; limited indications
**Phase III evidence**	Standard comparator	ReSTORE trial demonstrated non-inferiority to caspofungin for day-14 global cure and day-30 mortality, with similar safety; earlier blood culture clearance was numerically faster with rezafungin
**Common adverse effects**	Elevated liver enzymes, infusion reactions, hypokalaemia, fever, phlebitis	Similar safety profile to caspofungin; infusion-related reactions and gastrointestinal effects uncommon

## Conclusion

Rezafungin enabled successful outpatient treatment of multidrug-resistant *C. auris* sternal wound infection following complex cardiac surgery. Once-weekly dosing may offer practical advantages and reduce healthcare resource utilization in selected patients requiring prolonged antifungal therapy [12].

## Abbreviations

CLSI: Clinical and Laboratory Standards Institute
